# Lymphocyte Redox Imbalance and Reduced Proliferation after a Single Session of High Intensity Interval Exercise

**DOI:** 10.1371/journal.pone.0153647

**Published:** 2016-04-20

**Authors:** Rosalina Tossige-Gomes, Karine Beatriz Costa, Vinícius de Oliveira Ottone, Flávio de Castro Magalhães, Fabiano Trigueiro Amorim, Etel Rocha-Vieira

**Affiliations:** 1 Sociedade Brasileira de Fisiologia, Programa Multicêntrico de Pós-graduação em Ciências Fisiológicas, Universidade Federal dos Vales do Jequitinhonha e Mucuri, Diamantina, Minas Gerais, Brazil; 2 Departamento de Educação Física, Faculdade de Ciências Biológicas e da Saúde, Universidade Federal dos Vales do Jequitinhonha e Mucuri, Diamantina, Minas Gerais, Brazil; 3 Faculdade de Medicina, Universidade Federal dos Vales do Jequitinhonha e Mucuri, Diamantina, Minas Gerais, Brazil; Universitatsklinikum Freiburg, GERMANY

## Abstract

This study investigated whether an acute session of high-intensity interval training (HIIT) is sufficient to alter lymphocyte function and redox status. Sixteen young healthy men underwent a HIIT session on a cycloergometer, consisting of eight bouts of 1 min at 90–100% of peak power, with 75 seconds of active recovery at 30 W between bouts. Venous blood was collected before, immediately after, and 30 minutes after the HIIT session. In response to *Staphylococcus aureus* superantigen B (SEB) stimulation, lymphocyte proliferation decreased and the IL-2 concentration increased after the HIIT session. However, the HIIT session had no effect on lymphocyte proliferation or IL-2 response to phytohemagglutinin stimulation. The HIIT session also induced lymphocyte redox imbalance, characterized by an increase in the concentration of thiobarbituric acid reactive substances and a decrease in the activity of the antioxidant enzyme catalase. Lymphocyte viability was not affected by the HIIT session. The frequencies of CD25^+^ and CD69^+^ T helper and B lymphocytes in response to superantigen stimulation were lower after exercise, suggesting that superantigen-induced lymphocyte activation was reduced by HIIT. However, HIIT also led to a reduction in the frequency of CD4^+^ and CD19^+^ cells, so the frequencies of CD25^+^ and CD69^+^ cells within the CD4 and CD19 cell populations were not affected by HIIT. These data indicate that the reduced lymphocyte proliferation observed after HIIT is not due to reduced early lymphocyte activation by superantigen. Our findings show that an acute HIIT session promotes lymphocyte redox imbalance and reduces lymphocyte proliferation in response to superantigenic, but not to mitogenic stimulation. This observation cannot be explained by alteration of the early lymphocyte activation response to superantigen. The manner in which lymphocyte function modulation by an acute HIIT session can affect individual immunity and susceptibility to infection is important and requires further investigation.

## Introduction

Strenuous prolonged continuous aerobic exercise is associated with an increased susceptibility to infection [1–3], a large systemic inflammatory response [4] and a transient impairment in immune competence [5]. Cell-mediated immunity is recognized as an important arm of the immune system to clear infections. Adequate lymphocyte activation by antigens, which leads to cell proliferation and cytokine secretion, plays a critical role in the development of immune response against invading pathogens. Many studies have reported that lymphocyte proliferation and cytokine secretion, as well as cellular viability, are temporarily altered following acute bouts of prolonged continuous strenuous exercise [6]. Lymphocyte reduced proliferative response to phytohemagglutinin (PHA) stimulation after a 3-hour-long exercise, at 55% of maximum power output, was demonstrated by Nieman et al [7]. Similar results were observed by Chen et al [8] during a 1-hour-continuous run at 70% VO_2_max, although some reports have suggested that an exercise-induced decrease in lymphocyte proliferation is secondary to the alteration in the ratio of lymphocyte subsets [2, 9]. Also, mitogen-induced IL-2 and IFN-γ lymphocyte secretion is reduced after 1 hour of cycling at 70% VO_2_ peak [10]. Reduced mitogen-induced IFN-γ production was also observed after 2.5 hours of treadmill running at 75% VO_2_ max [11]. These findings support the idea of temporary immune function suppression after strenuous prolonged exercise.

Cellular redox imbalance is among the potential mechanisms responsible for exercise-induced immunosuppression associated with strenuous prolonged exercise. The generation of reactive oxygen species increases during exercise [12], which may overcome the mechanisms of antioxidant defense utilized by antioxidant enzymes, such as superoxide dismutase (SOD) and catalase (CAT). This imbalance can result in an oxidative damage to cellular components, compromising cell function and even leading to cell death. Exercise-induced redox imbalance in lymphocytes has been reported. Increased protein carbonyl content, a marker of oxidative damage to proteins, was observed in lymphocytes after 60 min of treadmill running at 80% of VO_2_ max [13]. Increased oxidative damage to lipids (thiobarbituric acid reactive substances, TBARS) in lymphocytes was also observed after prolonged strenuous exercise [14, 15]. However, to our knowledge, no study simultaneously evaluated the effect of strenuous exercise on lymphocyte function and redox status.

In recent years, high intensity interval training (HIIT) has gained popularity as a training modality for non-athletes, partially because of its low-volume characteristic. A HIIT session is characterized by a relatively brief intermittent exercise performed with an all-out effort or at an intensity close to that which elicits peak oxygen uptake (≥90% of VO_2_max) [16]. A HIIT session is relatively brief, consisting of approximately 10 min of intense exercise within a complete training session that lasts about 30 min. In this situation, the total weekly exercise and training time is reduced, compared to the current public health guidelines [17]. HIIT imposes higher and different metabolic, endocrine and inflammatory demands [18, 19] than work-matched continuous exercise. Similar to conventional endurance training, HIIT is sufficient to improve fitness and to induce beneficial metabolic adaptations [20, 21]. It is also beneficial for the improvement of the cardiorespiratory fitness in a range of populations, including those with coronary artery disease or congestive heart failure, as well as those with obesity [22, 23]. In this context, HIIT can be an alternative time-efficient intervention to improve metabolic health and reduce the risk of chronic diseases [24].

Despite its growing popularity, investigations regarding the effects of an acute HIIT session on lymphocyte function and redox status are lacking. Only one study examined the effect of an acute HIIT session on lymphocyte redox status to date. Fischer et al. [25] observed an increase in the SOD, CAT and glutathione peroxidase (GPX) activities in lymphocytes following a HIIT session. However, neither lymphocyte oxidative stress markers nor T cell functions were evaluated. A single HIIT session is sufficient to promote oxidative damage to plasma components, demonstrated as an increase in TBARS levels after exercise [25, 26], which can potentially compromise leukocyte functions. Therefore, this study investigated the effect of an acute HIIT session on the response of lymphocytes to a superantigenic and a mitogenic stimulation (proliferation and cytokine secretion) and on the redox status of these cells (reduced glutathione—GSH and TBARS content, SOD and CAT activity). We hypothesized that an acute HIIT session is sufficient to promote lymphocyte redox imbalance, thus resulting in reduced proliferation and cytokine secretion.

## Methods

### Subjects

Sixteen non-smoker, young, male subjects were selected to participate in this study. Volunteers were recruited from the local community based on the following criteria: not being engaged in a regular exercise program, not using anti-inflammatory drugs or being immunized seven days before the study, and not using anti-oxidant supplements. None of the subjects self-reported neuromuscular or musculoskeletal injuries and heart, lung or autoimmune diseases. All the individuals selected for the study were considered able to perform exercise, based on Par-Q (*Canada´s physical activity guide to healthy active living*) [27] and coronary risk factor questionnaires [28].

The Internal Review Board of the Universidade Federal dos Vales do Jequitinhonha e Mucuri approved the study. All volunteers signed an informed consent form, thereby agreeing to participate in the study.

### Study design

This study was divided into two parts. The first part (study #1) was designed to evaluate the effect of an acute HIIT session on lymphocyte function (proliferation and cytokine secretion in response to superantigen or mitogen stimulation) and its redox status, and enrolled 10 volunteers. In the second study (study #2), the effect of a HIIT session on the viability and the activation marker expression of lymphocyte subpopulations was evaluated, and six volunteers participated in this part of the study.

Volunteers were asked to refrain from strenuous physical activity for 24 hours, caffeine consumption for 48 hours, alcoholic beverages for 24 hours, topical corticosteroid or aspirin use for 48 hours, and systemic antihistamines or corticosteroid use for 1 week prior to the exercise sessions. Participants were also asked to sleep eight hours the night before the exercise sessions. In the morning of the tests, volunteers were asked to eat a standardized breakfast based on the American College of Sports Medicine [29], which recommends the intake of 200 to 300 g of carbohydrates before vigorous exercise. The intake of 500 ml of water two hours before the test was also recommended [29].

### Pre-HIIT measurements

Forty-eight hours before the HIIT session, volunteers underwent anthropometric measurements and determination of maximum oxygen consumption (VO_2_ peak) and maximum power output.

In the study #1, Balke cycle ergometer (Ideal, MMX, Brasil) protocol was used to estimate VO_2_ peak [30] and maximal power output. Briefly, the protocol consisted of cycling at a 25 W initial power, which was increased by 25 W every 2 minutes until the fatigue of the volunteers. The test was completed when subjects could no longer maintain the required power, despite verbal encouragement. The greatest power value obtained by each subject was registered, and the VO_2_ peak was estimated using the equation [31]:

$$VO_{2}peak\;\left(O_{2}ml^{-1}x\;min^{-1}\right)\;=\;200\;+\;\left[12\;x\;power\;\left(W\right)\right]/body\;mass\;\left(kg\right)$$

In the study #2, subjects had a maximum exercise test conducted on an electronically braked cycle ergometer (Excalibur Sport, Lode, USA) using the ramp protocol [32]. The power was increased every 60s at an individual rate based on the subjects’ exercise history questionnaire (Veterans Specific Activity Questionnaire) [33] to induce fatigue within 8 to 12 min. The test was completed when subjects could no longer maintain the required power, despite verbal encouragement. The greatest power value obtained by each subject was registered, and the VO_2_ peak was estimated using the ACSM [34] equation:

$$VO_{2}peak\;\left(O_{2}ml^{-1}\;x\;min^{-1}\right)\;=\;10.8\;x\;\left[work\;rate\;\left(kg\;x\;min^{-1}\right)\right]\;/\;body\;mass\;\left(kg\right)\;+\;7$$

In both situations, tests were conducted in an environmental chamber at 22°C and 60% relative humidity.

### HIIT session

The HIIT session was performed between 7h30 and 8h30 AM in an environmental chamber (22°C, 60% relative air humidity). Exercise was performed on an cycloergometer (Moviment, BM 2800 PRO, Brasil—study #1; Excalibur Sport, Lode, USA—study #2) and consisted of eight bouts of 1 min at 100% (study #1) or 90% (study #2) of peak power, intercalated with 75s-intervals of active recovery at 30 W. Volunteers cycled for 2 minutes at 30 W before and after the HIIT session.

Before (pre-ex), immediately after (post-ex) and 30 minutes after exercise (30 min-post), blood samples (approximately 20 ml) were collected from the antecubital vein and transferred to heparinized vacuum tubes (Vacutainer; Becton Dickinson, USA) for separation of peripheral blood mononuclear cells (PBMC) and further analysis of lymphocyte redox status and function.

### Study #1

#### Lymphocyte proliferative response

PBMC were isolated from heparinized blood by centrifugation in Histopaque^®^ 1077 (Sigma, USA) as described by Bicalho et al. [35]. Then, 1x10^7^ cells/ml were suspended in phosphate-buffered saline (PBS, 0.05 M, pH 7.4) with 0.1% bovine serum albumin (PBS/BSA 0.1%) and labeled with 5,6-Carboxyfluorescein diacetate N-succinimidyl ester (CFSE– 10 μM) (Sigma, USA) as described by Lyons et al. [36]. Cells were washed and suspended in RPMI 1640 (Sigma, USA) and supplemented with L-glutamine (2 mM), antibiotic/antimycotic solution (100 UI/ml penicillin G, 100 μg/ml streptomycin and 2.5 μg/ml fungizone) (Sigma, USA), and fetal calf serum (10%) (Gibco, USA). Cells (5x10^5^) were stimulated with the *Staphylococcus aureus* superantigen B (SEB, 100 ng/ml) (Sigma, USA) or phytohemagglutinin (PHA, 0.001 mg/ml) (Sigma, USA) for 5 days at 37°C and 5% CO_2_. After the culture period, cells were collected, washed in PBS and fixed with 2% paraformaldehyde for posterior analysis by flow cytometry.

**Fig 1:**
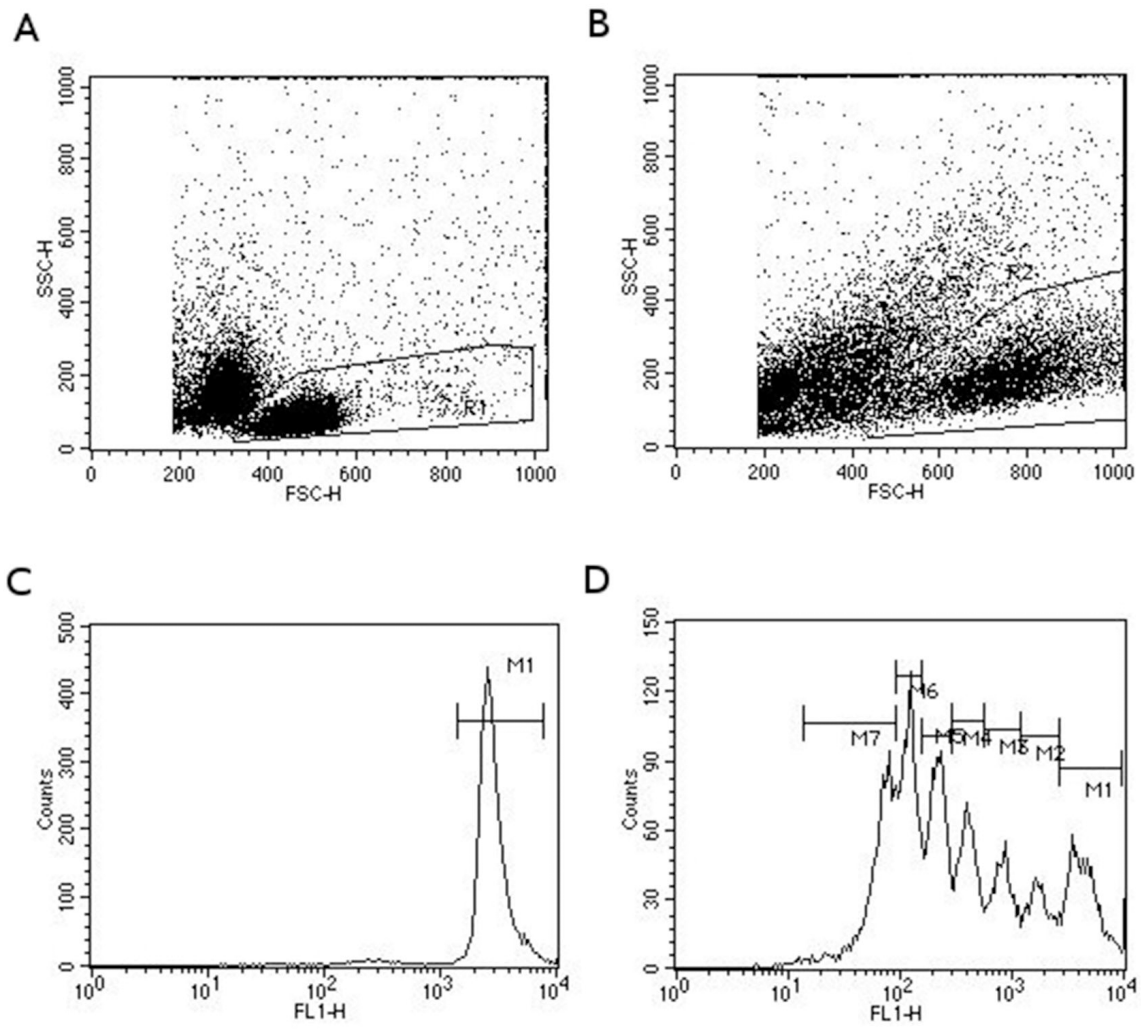
Analysis strategy used to evaluate the proliferative response of lymphocytes by CFSE-fluorescence decay in a flow cytometer. The profile of size (FSC) and granularity (SSC) of non-stimulated (A) and SEB-stimulated cells (B). FL1 fluorescence (CFSE) histograms for cells delimited in graph A (C) and B (D). M1 indicate the peak of quiescent cells, and M2 to M7 of cells that have passed by one or more mitotic events.

The proliferative response of lymphocytes was evaluated by the decay of CFSE fluorescence using the FACScan (Becton Dickinson, San Jose, CA, USA) flow cytometer equipped with a blue argon laser (488 nm) and a 530/30 nm band pass filter. Fifty thousand events in the region of small lymphocytes and blast cells were acquired for posterior analysis of non-stimulated (Fig 1A) and stimulated cultures (Fig 1B). Acquired data were analyzed using the Cell Quest software (Becton Dickinson). Events in R1 were analyzed for CFSE fluorescence in histograms (Fig 1C and 1D). The M1 region was defined as CFSE-stained cells derived from non-stimulated cultures, which represents the peak of quiescent cells (Fig 1C). M2 to M7 regions were defined according to the peaks of different CFSE intensities in stimulated cultures (Fig 1D).

The proliferative index was then calculated from the data of the histograms, considering that two cells with a given CFSE intensity emerged from the mitosis of a single cell possessing a CFSE intensity immediately superior, using the following formula [37]:

$$IP=\;\frac{100-Y}{Y},\;\text{where}\;Y\;\left(\%\right)=X0+\;\frac{\text{X}1}{2}+\;\frac{\text{X}2}{4}+\;\frac{\text{X}3}{8}+\;\frac{\text{X}4}{16}+\frac{\text{X}5}{32}+\;\frac{\text{X}6}{64}+\frac{\text{X}7}{128}$$

X0 represents the percentage of cells that did not divide (located in M1), and X1 to X7 represents the peak of gradual division (percentage of cells on M2 to M7).

#### Lymphocyte cytokine secretion

PBMC (5x10^5^ cells) were stimulated with SEB (100 ng/ml) or PHA (0.001mg/ml) for 24 hours at 37°C and 5% CO_2_. Culture supernatant was then collected, centrifuged (200 *x*g, 25°C, 10 min) and stored at -80°C for posterior analysis. IL-2, IL-4 and IFN-γ concentrations in culture supernatant were determined by sandwich ELISA, using the ELISA DuoSet kit (R&D Systems, USA), according to the manufacturer instructions.

#### Lymphocyte redox status

The redox status of lymphocytes was determined by the measurement of the TBARS concentration, GSH content and activity of the antioxidant enzymes SOD and CAT. PBMC (1x10^7^ cells/ml), suspended in PBS (0.015 M, pH 7.2–7.4), were freeze-thawed three times to induce cell disruption. Cell lysate was centrifuged at 10,000 *xg* for 15 min. The supernatant was used to determine the activity of the enzymes SOD and CAT and the TBARS measurement. GSH content was determined in intact PBMC. Because PBMCs are composed predominantly by lymphocytes we assumed that PBMC redox status had a predominant contribution of the lymphocyte redox status.

The TBARS concentration was determined during an acid-heating reaction, as described by Ohkawa et al [38]. Briefly, cell lysate (0.2ml) was added to sodium dodecyl sulfate (8.1%, 0.1 ml), acetic acid (0.25 ml, 2.5 M, pH 3.4), and 0.8% thiobarbituric acid (0.25 ml, Sigma, USA). Samples were heated in boiling water (90°C) for 90 min and centrifuged for 5 min at 5,000 *xg* (Jouan BR4i, Thermo Fisher Scientific, EUA). Supernatant was used for TBARS determination at 532 nm (Spectra Max 190, Molecular Devices, Sunnyvale, CA, USA). The values were compared with a standard curve, which was made with known concentrations of malondialdehyde (1,1,3,3-tetramethoxypropane) (Sigma, USA). The amount of malondialdehyde (MDA) produced was interpreted as the TBARS levels and indicates the degree of oxidative stress. The results are expressed as MDA equivalents per 10^7^ cells. Measurements were performed in duplicate.

The assay to determine SOD (EC 1.15.1.1) activity was performed according to Srivastava et al. [39] with some modifications. In brief, cell lysate supernatant (0.25 ml) was added to sodium phosphate buffer (50 mM, pH 8.2, 37°C) containing 1 mM diethylenetriamine pentaacetic acid (DTPA, Sigma, USA). The samples and buffer were heated up to 37°C for 3 min. The reaction was initiated by addition of 0.2 mM pyrogallol (Sigma, USA). Absorbance was determined at 420 nm during 4 minutes (Spectra Max 190, Molecular Devices, Sunnyvale, CA, USA). SOD activity was calculated as units per 10^7^ cells, and one unit of enzyme was considered as being the amount that caused 50% inhibition of pyrogallol autoxidation. Measurements were performed in duplicate.

Catalase (EC 1.11.1.6) activity was measured in the supernatants, as described by Nelson and Kiesow [40]. Briefly, H_2_O_2_ (0,3M) was added as a substrate to cell lysate supernatant (0.25 ml) in potassium phosphate buffer (50 mM, pH 7.0), giving a final H_2_O_2_ concentration of 2 mM. The reaction proceeded for 1 min at room temperature. Decomposition of H_2_O_2_ by catalase was noted by the change in absorbance at 240 nm (ΔE) during 1 minute (Spectra Max 190, Molecular Devices, Sunnyvale, CA, USA). This procedure avoids the possible interference associated with glutathione peroxidase activity, because the necessary cofactors are not present in the reaction media. Catalase activity was expressed as millimoles of H_2_O_2_ decomposed per minute per 10^7^ cells (ΔE/min^-1^/10^7^ cells). Measurements were performed in duplicate.

**Fig 2:**
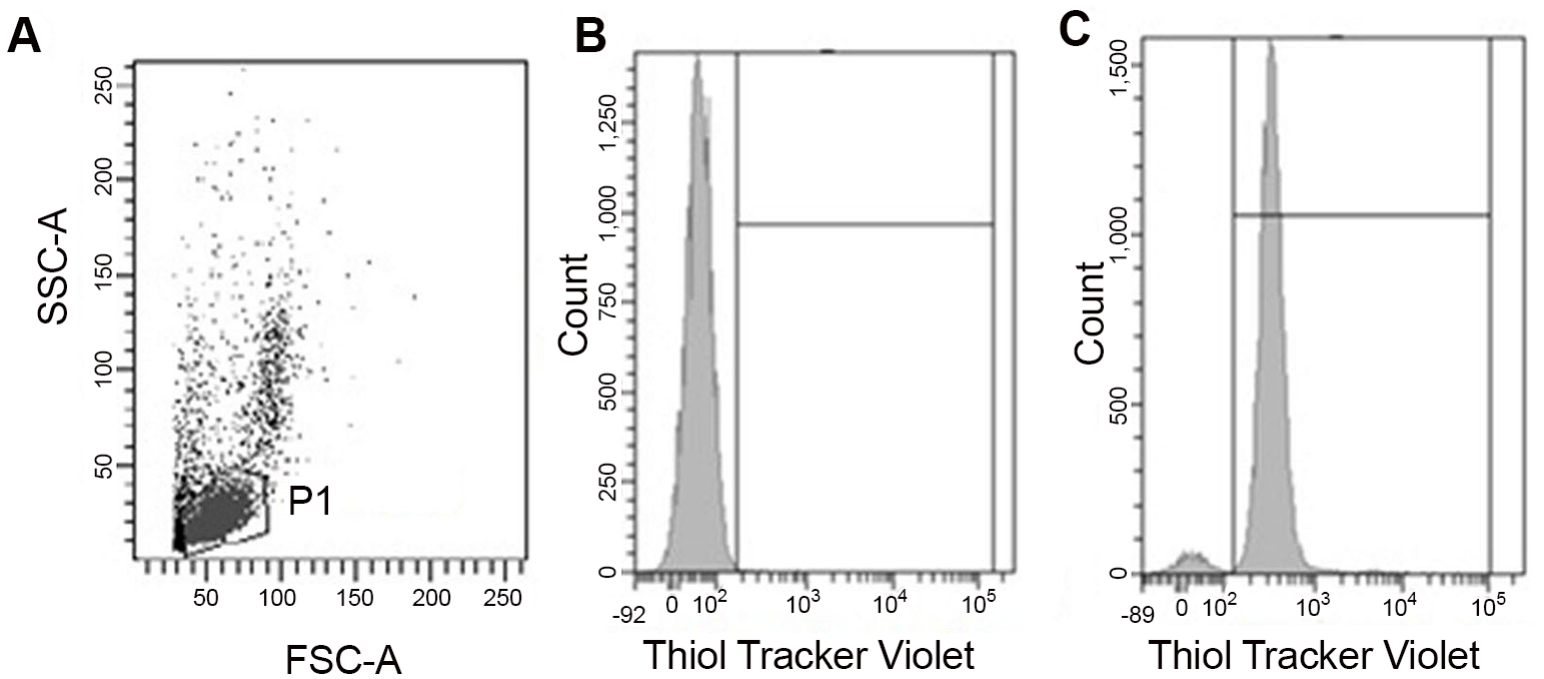
Analytic strategy used to evaluate lymphocyte GSH content by flow cytometry. Profile of size (FSC) and granularity (SSC) (A). Fluorescence intensity histograms of non-labeled (control) (B) and Thiol Tracker-labeled cells (C).

Reduced glutathione (GSH) content was determined in intact PBMC by flow cytometry using the Thiol Tracker Violet^®^ (Molecular Probes, USA), according to the method of Mandavilli and Janes [41]. Briefly, the PBMC suspension (1x10^6^ cells) was stained with Thiol Tracker (2 μM) for 30 min at 37°C. Cells were then centrifuged at 200 *xg* for 10 min and suspended in PBS (0.015M, pH 7.2–7.4). The GSH content was estimated on a FACSCanto II (Becton Dickinson) flow cytometer equipped with a 405 nm excitation laser and a 550/50 nm band pass filter. Thirty thousand events in the lymphocyte region were acquired. Data were analyzed using the FACS Diva v.6.1.3 software (Becton Dickinson). Events in P1 were analyzed for thiol tracker fluorescence in histograms (Fig 2B and 2C). Mean fluorescence intensity of the thiol-tracker-stained cells was used to estimate the GSH content (Fig 2C). Non-stained cells were used to establish the threshold for the thiol tracker fluorescence emission (Fig 2B).

### Study #2

#### Analysis of cell death by apoptosis

The assay was performed using the Apoptosis Detection kit FITC-Annexin V (BD Pharmingen, USA) based on the method proposed by Vermes et al. [42]. Briefly, 2x10^5^ cells were stained with anti-anexin V-FITC (1 μl) and propidium iodide (PI, 1 μl) in 8 μl of binding buffer (provided by the manufacturer) during 15 min in the dark at room temperature. Then, 80 μl of binding buffer was added, and the cells were washed twice (200 *xg*, 7 min, 4°C). Cells were suspended in PBS and analyzed by flow cytometry immediately after staining, using the FACScan flow cytometer equipped with a blue argon laser (488 nm) and 530/30 nm and 586/42 nm band pass filters. Twenty thousand events in the lymphocyte region were acquired. The frequency of necrotic cells (marked by propidium iodide only), as well as cells in recent (marked only by anti-anexin) and late apoptosis (marked by anti-anexin and propidium iodide) were determined.

#### Activation markers expression by lymphocyte subpopulations

Surface expression of early activation markers—CD25 and CD69 –by major lymphocyte subpopulations—CD4^+^, CD8^high^ and CD19^+^ cells—in response to SEB stimulation was evaluated. PBMC were stimulated with SEB (100 ng/ml) for 18 hours at 37°C and 5% CO_2_. Cells were then collected, washed in PBS, and labeled with monoclonal antibodies (mouse anti-human) against surface molecules as previously described. The following fluorochrome-conjugated antibody cocktail was used: CD69-FITC (FN50) + CD4-PE (RPA-T4) + CD8-PECy5 (MOPC-21); CD25-FITC (M-A251) + CD4 PE (RPA-T4) + CD8 PECy5; and CD19-PE (HIB19) + CD69 FITC. Rat anti-mouse IgG1 FITC (A85-1), rat anti-mouse IgG2a+b PE (X57), and rat-anti mouse IgG2a+b PerCP (X57) were used as isotype controls. All antibodies used were branded BD Pharmingen.

The frequency of CD4^+^, CD8^high^, CD19^+^, CD4^+^CD25^+^, CD4^+^CD69^+^, CD8^high^CD25^+^, CD8^high^CD69^+^ and CD19^+^CD69^+^ lymphocytes was evaluated using the FACSCanto II flow cytometer equipped with a blue argon laser, 530/30-nm and 585/42-nm band pass filters, and 556 nm, 655 nm and 670-nm long pass filters. Thirty thousand events in the lymphocyte region were acquired, and data were analyzed using the FACS Diva v.6.1.3 software.

### Statistical analysis

The Statistica (v10.0, StatSoft, Inc) software was used for statistical analysis. P values ≤ 0.05 were considered statistically significant. Data are reported as the mean ± SE. The Shapiro—Wilk test was used to evaluate the normalcy of the data. Since the dependent variables were normally distributed, parametric tests were used for statistical analysis. Student’s t-test was used to compare data from studies #1 and #2. A one-way analysis of variance (ANOVA one-way with repeated measures) was used to evaluate the HIIT effect on redox status parameters (TBARS, GSH, CAT and SOD activity) and cell viability. A two-way analysis of variance (ANOVA two-way with repeated measures) was used to evaluate the HIIT effect on SEB-induced proliferation, cytokine production and activation marker expression. The significance of the difference between the means was determined by the Tukey post hoc test. To check for size differences between pre, immediate and 30-min post-exercise periods, the magnitude of the *post-hoc* effects (ƒ) and the statistical power were calculated using G*3 Power.

## Results

The physical and physiological data of the volunteers who participated in both studies are shown in Tables 1 and 2. Except for maximum power output, no difference was observed between volunteers of studies #1 and #2 for the following variables: age and body mass index (BMI), VO_2_peak, maximum heart rate and maximum rate of perceived exertion achieved during the maximum test (Table 1). Despite the difference in maximum power output, there was no difference between the studies with regard to effort during the HIIT session, as demonstrated by mean heart rate (HR) and mean rate of perceived exertion (RPE) during the HIIT bouts and active recovery intervals (Table 2). The mean HR achieved during the HIIT session by the volunteers corresponded to 85% of the peak HR observed during the Balke test in study #1. In study #2, the mean HR achieved during the HIIT session corresponded to 86% of the peak HR found during the ramp protocol. These data indicate that the HIIT sessions were performed at high intensity in both studies.

**Table 1:** Physical and physiological characteristics of volunteers.

Characteristics	Study #1	Study #2	P value
Age (years)	23.7 ± 1.1	21.3 ± 1.8	0.24
BMI (kg/m^2^)	21.3 ± 0.8	21.9 ± 0.7	0.63
VO_2_peak (mlO_2_.kg^-1^.min^-1^)	33.6 ± 1.2	36.6 ± 1.8	0.17
Peak power (W)	172.3 ± 7.0	233.8 ± 19.8	< 0.01
Peak RPE	19.0 ± 1.0	19.0 ± 1.0	0.14
Peak HR (bpm)	169.0 ± 7.0	182.0 ± 5.0	0.58

Data expressed as mean ± SE. BMI = body mass index, RPE = rate of perceived exertion, HR = heart rate, bpm = beats per minute. Means compared using Student’s t-test. Study #1 N = 10, study #2 N = 6.

**Table 2:** Heart rate and perceived exertion during the HIIT sessions.

	Study #1	Study #2	P value
Bouts mean HR (bpm)	144.0 ± 6.0	157.0 ± 5.0	0.16
Recoveries mean HR (bpm)	117.0 ± 5.0	120.0 ± 5.0	0.69
Bouts mean RPE	13.0 ± 1.0	13.0 ± 1.0	0.65
Recoveries mean RPE	11.0 ± 1.0	10.0 ± 1.0	0.25

Data expressed as mean ± SE. HIIT = high intensity interval training, bpm = beats per minute, HR = heart rate, RPE = rate of perceived exertion. Means compared using Student’s t-test. Study #1 N = 10, study #2 N = 6.

### Study #1—HIIT effect on lymphocyte function and redox status

**Fig 3:**
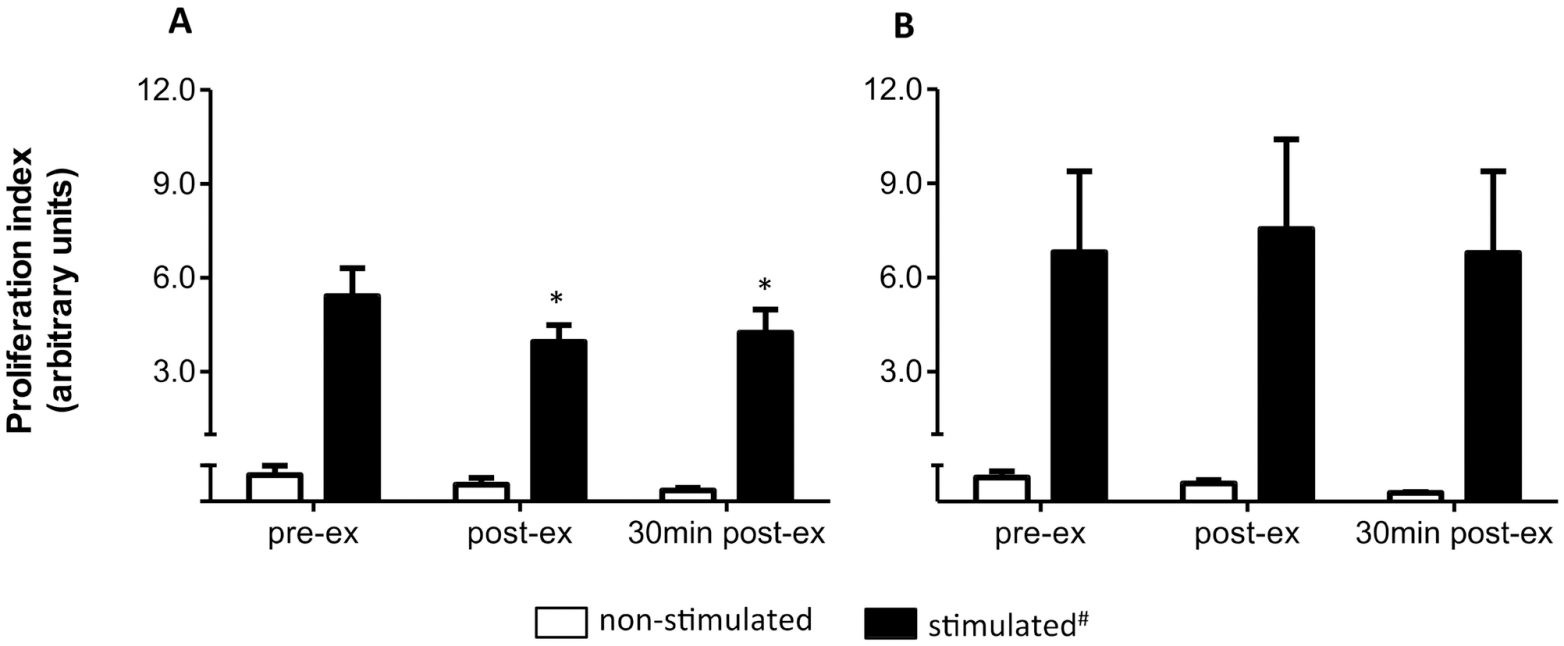
HIIT effect on lymphocyte proliferative response. **(A)** SEB-stimulated cells. **(B)** PHA-stimulated cells. Data shown as mean ± SE. ^#^P < 0.05, compared to non-stimulated cells, in all moments. *P < 0.05, compared to pre-ex, Anova-two way, *Tukey post-hoc*. pre-ex = before exercise, post-ex = immediately after exercise, 30 min post-ex = 30 min after exercise. N = 10.

Fig 3 shows the effect of the HIIT session on SEB- and PHA-induced proliferative response of lymphocytes. A time *versus* stimulus interaction was observed with SEB (Fig 3A), but not with PHA stimulation (Fig 3B). The proliferative index decreased (P = 0.02, power = 0.8, ƒ = 0.5) immediately and 30 minutes after the exercise session in response to SEB stimulation. No effect of the HIIT session on the lymphocyte proliferative response to PHA stimulation was observed.

The lower proliferation index may be due to a reduction in the number of cells that were committed to proliferation. The percentage of cells located in M1 (Fig 1)–cells that did not divide after 5 days of stimulation—increased almost 35% (P = 0.05, ANOVA one way, Tukey *post hoc*) immediately after exercise (16.3 ± 13.2%), compared with pre-exercise values (12.0 ± 8.2%) for SEB-stimulated cells, suggesting that, at least in part, reduced proliferation can be a consequence of fewer cells committed to proliferation. In accordance with the absence of an effect of HIIT on lymphocyte proliferative response to PHA stimulation, the frequency of cells located in M1 upon PHA stimulation was not different (P = 0.9, P = 0.05, ANOVA one way, Tukey *post hoc*) when pre-exercise (11.5 ± 9.5%) to post-exercise (11.4 ± 9.0%) and 30 min post-exercise (11.8 ± 10.7%) values were compared.

**Fig 4:**
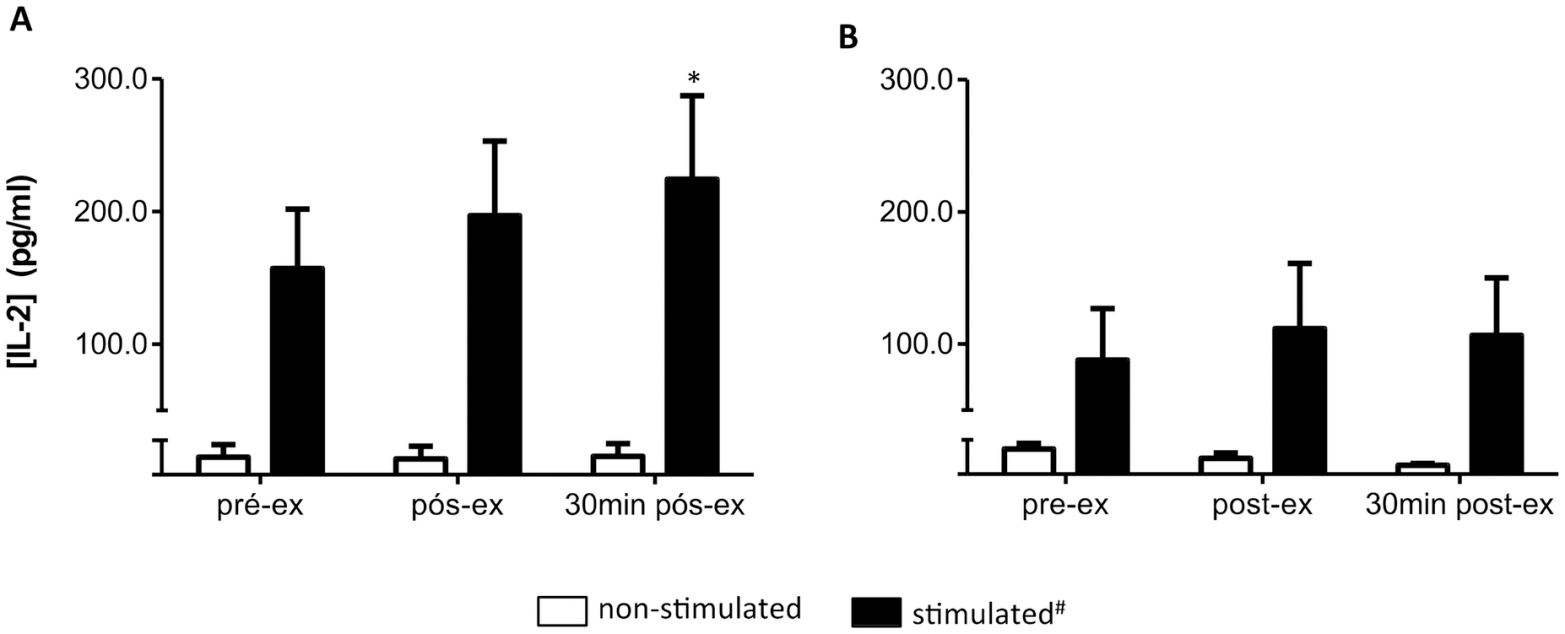
HIIT effect on IL-2 concentration in culture supernatant. **(A)** SEB-stimulated cells. **(B)** PHA-stimulated cells. Data shown as mean ± SE. #P < 0.05, compared to non-stimulated cells, in all moments. *P < 0.05, compared to pre-ex. Anova-two way, Tukey *post-hoc*. pre-ex = before exercise, post-ex = immediately after exercise, 30 min post-ex = 30 min after exercise. N = 10.

After the HIIT session, the IL-2 lymphocyte secretion was altered in response to SEB, but not to PHA stimulation. Once again, a time *versus* stimulus interaction (P = 0.03) was observed with SEB stimulation. An increased IL-2 concentration in culture supernatant in response to SEB stimulation 30 min after exercise (P = 0.006, power = 0.6, ƒ = 0.4) is shown by the data in Fig 4A. No effect of exercise on the IL-2 concentration in culture supernatant of PHA-stimulated cells (P = 0.2) (Fig 4B) was seen. HIIT also did not affect IFN-γ secretion by SEB-stimulated cells (299.0 ± 87.6, 305.8 ± 40.0, and 413.5 ± 93.7 pg/ml for, pre-ex, post-ex, and 30 min-post exercise, respectively). IL-4 was not detected in culture supernatant.

**Fig 5:**
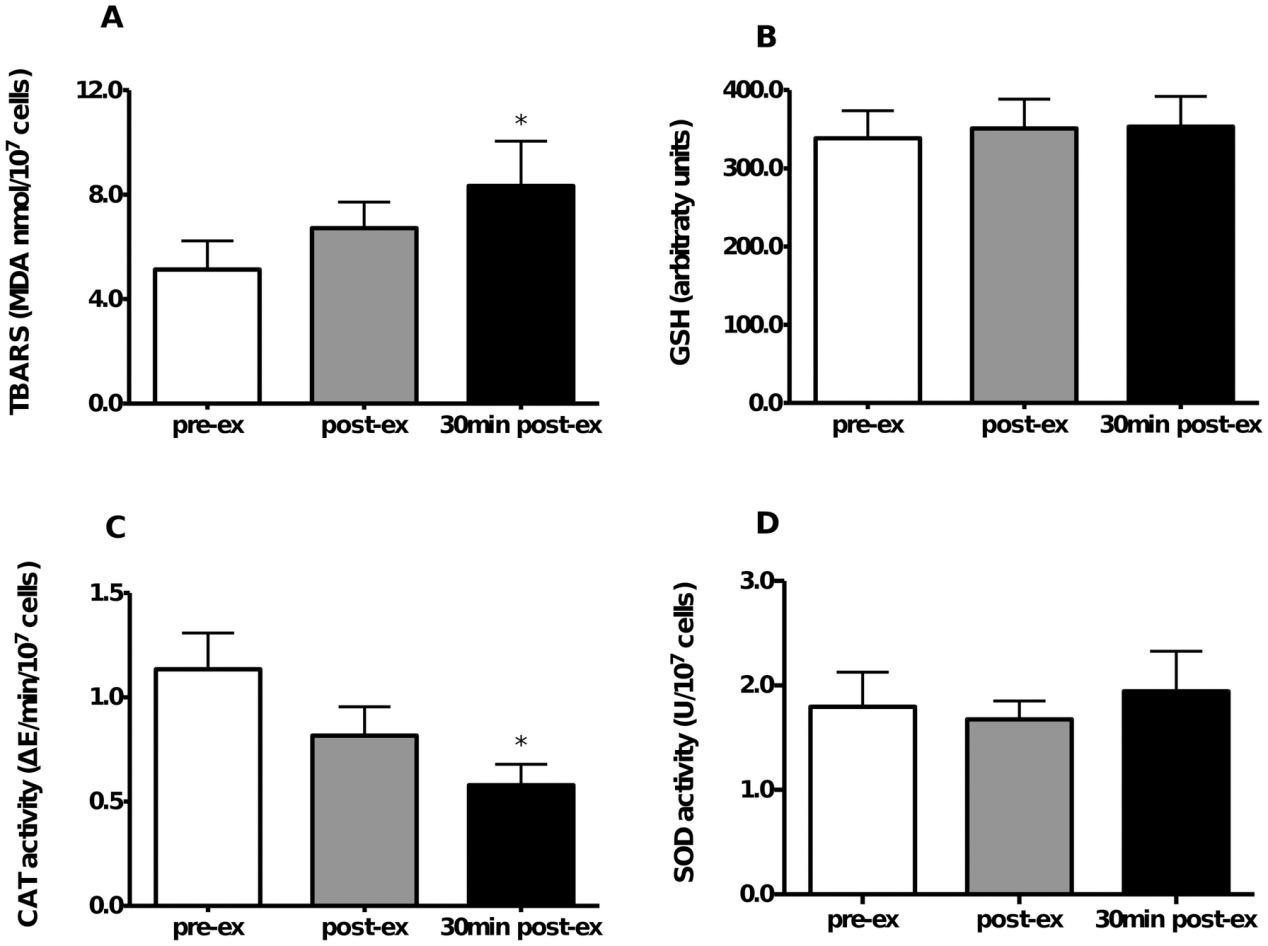
Modulation of lymphocyte redox status by HIIT. **(A)** TBARS concentration. **(B)** GSH content. **(C)** CAT activity. **(D)** SOD activity. *P < 0.05, compared to pre-ex, one-way Anova, Tukey *post hoc*. pre-ex = before exercise, post-ex = immediately after exercise, 30 min post-ex = 30 min after exercise. N = 10.

The HIIT effect on lymphocyte redox status is shown in Fig 5. TBARS were higher 30 min after exercise (P = 0.01, power = 0.7, ƒ = 0.7) (Fig 5A), whereas no effect of the HIIT session was observed on the GSH content (Fig 5B) and SOD activity (Fig 5D). However, lymphocyte CAT activity was lower 30 min after exercise (P = 0.03, power = 0.6, ƒ = 0.6) (Fig 5C). These data suggest that the HIIT session induced lymphocyte redox imbalance.

### Study # 2 –HIIT effect on lymphocyte viability and activation marker expression

Because we observed a reduced proliferative response of lymphocytes along with an increased IL-2 concentration in culture supernatant, which were accompanied by a redox imbalance, we performed the second study to evaluate the effect of the HIIT session on cellular viability and SEB-induced activation markers expression by lymphocyte subpopulations. As shown in Table 3, no HIIT effect was observed on the frequency of viable, apoptotic or necrotic cells.

**Table 3:** Effect of the HIIT session on lymphocyte viability.

	pre-ex	post-ex	30 min post-ex	P
viable cells (%)	76.9 ± 8.8	76.6 ± 9.9	75.6 ± 10.3	0.904
recent apoptosis (%)	22.3 ± 8.5	22.8 ± 9.7	23.5 ± 9.6	0.711
late apoptosis (%)	0.6 ± 0.4	0.5 ± 0.3	0.7 ± 0.6	0.329
necrosis (%)	0.2 ± 0.1	0.1 ± 0.0	0.2 ± 0.1	0.053

Data expressed as mean ± SE. Pre-ex = pre-exercise, post-ex = immediately after exercise, 30min post-ex = 30 min after exercise. Means compared using ANOVA one-way, Tukey *post hoc*. N = 6.

**Fig 6:**
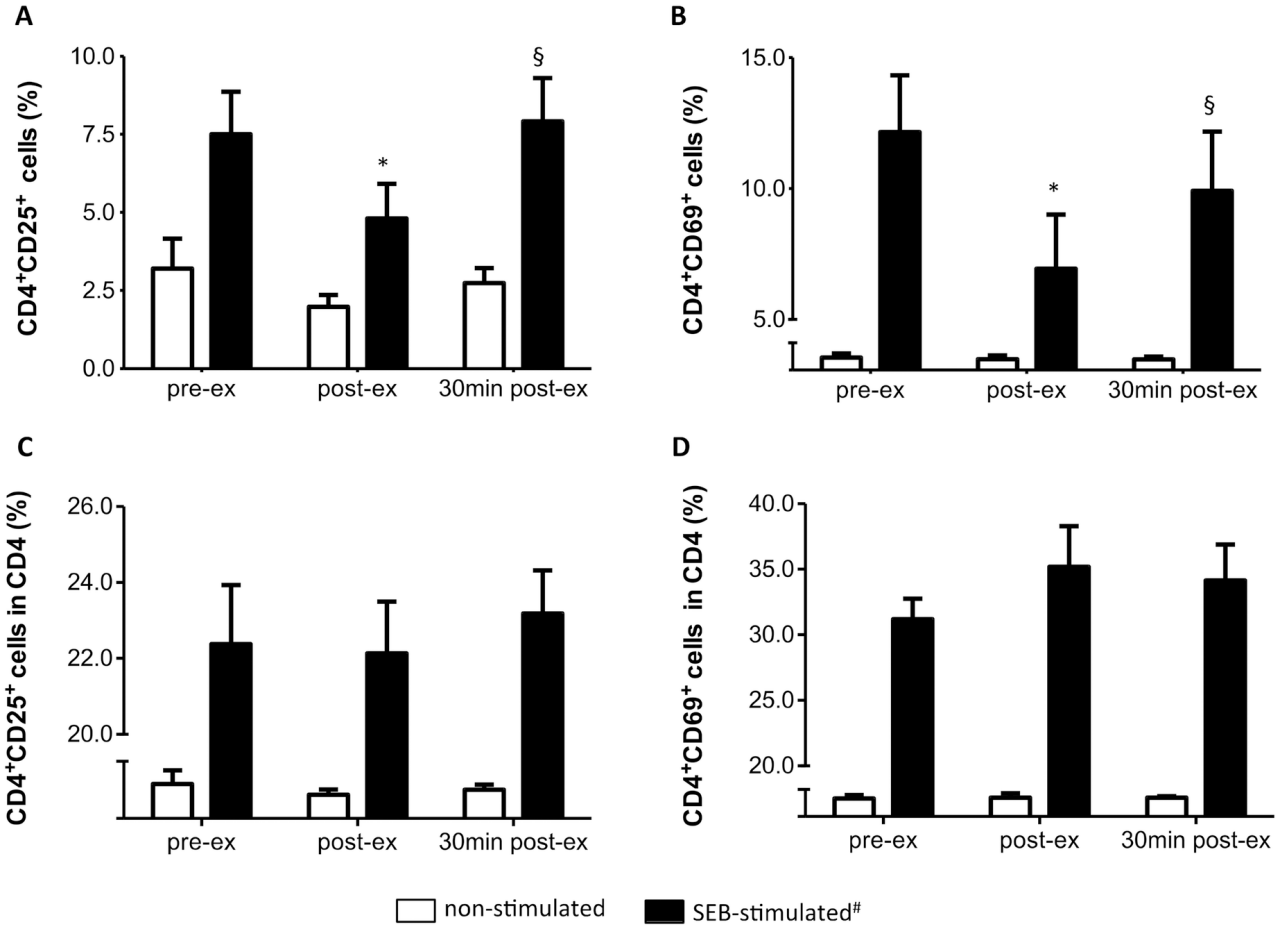
HIIT effect on TCD4 activation by SEB. **(A)** Frequency of CD4^+^CD25^+^ lymphocytes. **(B)** Frequency of CD4^+^CD69^+^ lymphocytes. **(C)** Frequency of CD4^+^CD25^+^ cells within the T helper (CD4^+^) cell subpopulation. **(D)** Frequency of CD4^+^CD69^+^ cells within the T helper (CD4^+^) cell subpopulation. Data shown as mean ± SE. # P <0.05, compared to non-stimulated cells in all moments. *P < 0.05, compared to pre-ex. ^§^P < 0.05, compared to post-ex. Two way Anova, Tukey *post-hoc*. pre-ex = before exercise, post-ex = immediately after exercise, 30 min post-ex = 30 min after exercise. N = 6.

The HIIT effect on SEB-induced TCD4 activation is shown in Fig 6. SEB-induced increase in the percentage of CD4^+^CD25^+^ (Fig 6A) and CD4^+^CD69^+^ cells (Fig 6B) was lower immediately after exercise (P = 0.005, power = 0.8, ƒ = 0.7 and P = 0.0004, power = 0.9, ƒ = 1.06, for CD4^+^CD25^+^ and CD4^+^CD69^+^ cells, respectively), suggesting a reduced TCD4 response to SEB after the HIIT session. However, the percentage of CD4^+^ cells was modified by exercise. Immediately after exercise, the CD4 percentage was 40% lower than pre-ex values (33.5 ± 20.6% and 20.0 ± 11.0%; pre- and post-ex, respectively, P = 0.006), although it returned to pre-ex levels 30 min after the exercise (27.3 ± 12.2%). Thus, the percentage of CD4^+^CD25^+^ (Fig 6C) and CD4^+^CD69^+^ cells (Fig 6D) within the CD4^+^ subpopulation was not modified by the HIIT session, which indicates that the reduced percentage of CD4^+^ activated cells was due to a reduction in the CD4^+^ cell number.

**Fig 7:**
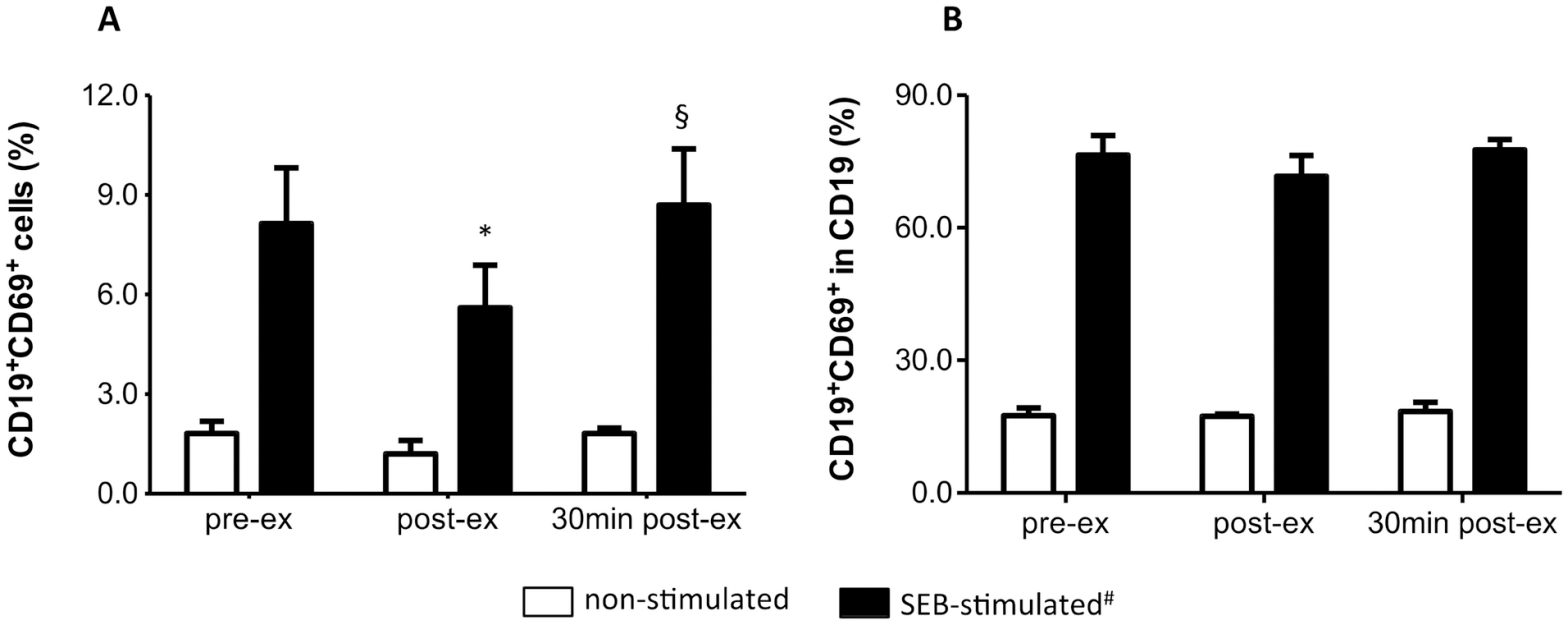
HIIT effect on B cell activation by SEB. **(A)** Frequency of CD19^+^CD69^+^ lymphocytes. **(B)** Frequency of CD19^+^CD69^+^ cells within the B (CD19^+^) cell subpopulation. Data shown as mean ± SE. ^#^P <0.05, compared to non-stimulated cells in all moments. *P < 0.05, compared to pre-ex; ^§^P < 0.05, compared to post-ex. Two-way Anova, Tukey *post-hoc*. pre-ex = before exercise, post-ex = immediately after exercise, 30 min post-ex = 30 min after exercise. N = 6.

A similar response was observed for B cell (CD19^+^) activation (Fig 7). SEB-induced increase in CD19^+^CD69^+^ cell frequency was lower immediately after exercise compared with pre-ex (P ≤ 0.01, power = 0.6 and ƒ = 0.6) (Fig 7A). However, exercise also promoted a decrease in CD19^+^ lymphocyte frequency immediately after exercise (10.2 ± 5.1% and 5.4 ± 1.9%, pre- and post-ex, respectively, P = 0.02), although it returned to pre-ex levels 30 min after exercise (10.9 ± 3.7%). No effect of exercise on B cell activation was observed, considering the percentage of CD19^+^CD69^+^ within the percentage of B cells (Fig 7B).

**Fig 8:**
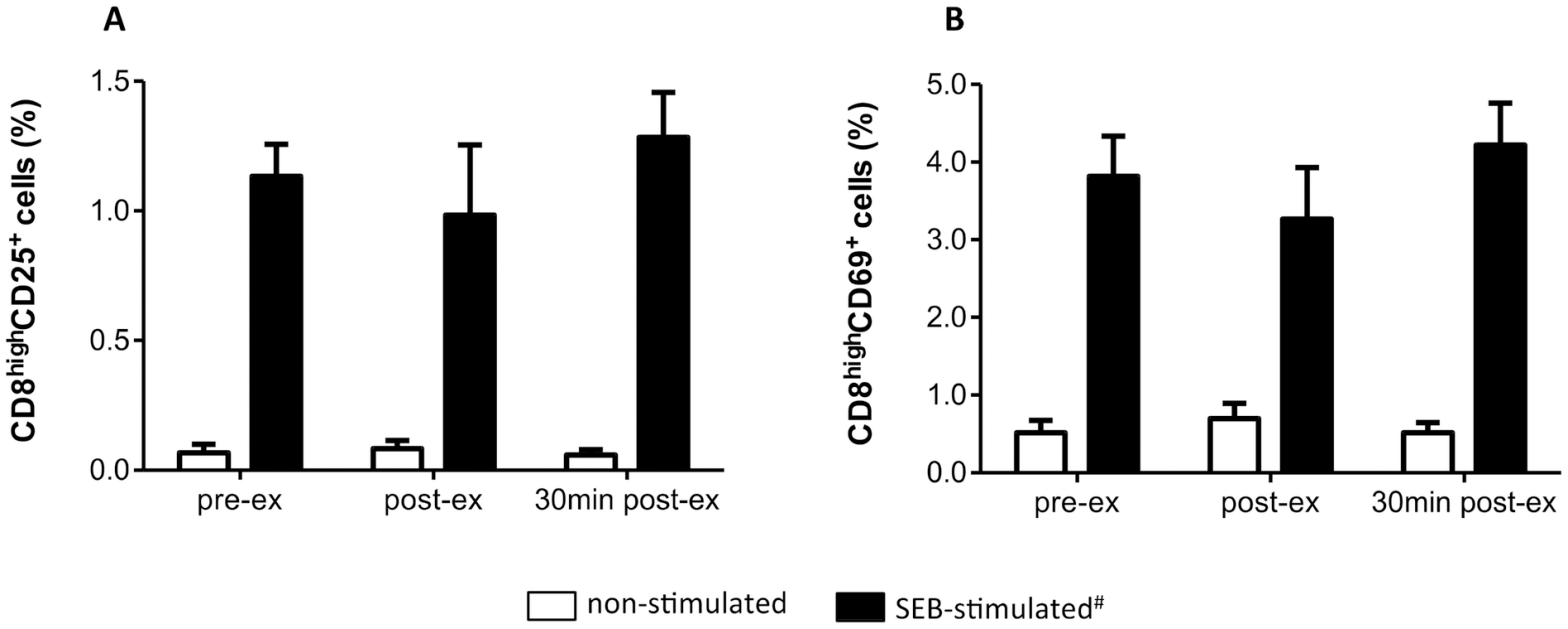
HIIT effect on cytotoxic T CD8 cell activation by SEB. **(A)** Frequency of CD8^high^CD25^+^ lymphocytes. **(B)** Frequency of CD8^high^CD69^+^ lymphocytes. Data shown as mean ± SE. pre-ex = before exercise, post-ex = immediately after exercise, 30 min post-ex = 30 min after exercise. N = 6.

No effect of the HIIT session on cytotoxic T cell (CD8^high^) frequency (19.2 ± 7.1% pre-ex, 16.9 ± 6.3% post-ex and 17.5 ± 5% 30 min post-ex, P = 0.36) was observed. No effect of exercise on SEB-induced activation marker expression by TCD8, CD25 (Fig 8A) and CD69 (Fig 8B) was observed.

## Discussion

In the present study, we investigated whether lymphocyte function and redox status are affected by an acute HIIT session. After the HIIT session, lymphocyte proliferative response to superantigen stimulation decreased, despite the higher IL-2 concentration in the culture supernatant. The HIIT session also induced a lymphocyte redox imbalance, which was observed by the higher TBARS levels and the lower catalase activity.

To our knowledge, this is the first report on simultaneous evaluation of the HIIT effect on lymphocyte function and redox status. Fischer et al. [25] previously observed an increase in lymphocyte SOD, CAT and GPX activities following a HIIT session, but neither the lymphocyte oxidative stress markers nor T cell function was evaluated. We now report elevated lymphocyte TBARS levels and a reduced CAT activity, clearly indicating a lymphocyte redox imbalance after HIIT. T cells usually control their intracellular redox balance through several cytosolic anti-oxidant systems, including the antioxidant enzyme CAT [43]. The reduced CAT activity observed may compromise the T cell’s ability to deal with reactive oxygen and/or nitrogen species (RONS), resulting in damage to cellular structures, as shown by the higher TBARS levels.

The maintenance of both intra- and extracellular reducing conditions is a prerequisite for the proper functioning of T lymphocytes. Several functions of lymphocytes are strongly regulated by redox status, including activation, proliferation and apoptosis [44]. *In vitro* studies on the effects of oxidative stress on T lymphocytes showed that RONS decrease antigen-specific T cell proliferation, but not mitogen-induced proliferation [44, 45]. Lymphocyte hyporesponsiveness to antigenic stimulation is partially recovered by CAT [44]. In accordance with these results, we observed that the HIIT session induced a redox imbalance along with a lower lymphocyte proliferative response to SEB, but not to PHA stimulation. It suggests a direct effect of HIIT-induced redox imbalance on antigen-specific lymphocyte proliferation, but not on the lymphocyte mitogen-activated response. Superantigens are a group of microbial proteins presented by MHC class II molecules to the TCR/CD3 complex, and their stimulation of T cells requires autologous or MHC class II matched antigen-presenting cells, similar to conventional antigens [46]. Because lymphocyte responses are antigen specific, assessment of *in vitro* cell responses to mitogens, such as PHA, does not necessarily provide a good model for the *in vivo* situation. Mitogens activate a high percentage of lymphocytes, making this a rather nonspecific method that might result in unrealistic large alterations in T cell function. Our data on the effect of an acute HIIT session on the lymphocyte proliferative response to SEB and PHA clearly illustrates this principle and shows how selection of proper lymphocyte stimuli can influence the outcome of an exercise immunology study. Our results also suggest that the HIIT effect on the lymphocyte superantigenic-specific proliferative response involves molecular components not engaged in the PHA-induced response.

The HIIT session also modified the SEB-induced IL-2 concentration in the culture supernatant, but it did not affect the IL-2 response to PHA stimulation. In contrast to the reduction in the proliferative response, the IL-2 concentration in the culture supernatant was increased after exercise in response to SEB stimulation. This observation may initially be contradictory becuse this cytokine is the main growth factor for antigen-stimulated lymphocytes [47]. However, the IL-2 concentration in the culture supernatant may not exactly reflect cytokine cell production because the cells in the culture can consume this cytokine. In this context, an increase in IL-2 in the culture supernatant may reflect a reduced consumption of this cytokine by the cultured cells, leading to the observed reduced cell proliferation.

The reduced proliferation induced by redox imbalance can result from a lower cellular viability (i.e., membrane permeability altered by lipid peroxidation), a lower degree of cell activation, or both because many components of signaling pathways are modulated by the cellular redox status [44]. To address these questions, we investigated the effect of the HIIT session on lymphocyte apoptosis and SEB-induced early activation marker expression. No effect of HIIT on the frequency of apoptotic cells prior to stimulation was observed, thus suggesting that the decrease in proliferative response to SEB stimulation after the HIIT session was not due to compromised cell viability. Although Cemerski et al. [44] demonstrated that lymphocyte hyporesponsiveness to antigenic stimulation upon oxidative stress was not due to increased apoptosis, we did not evaluate apoptosis upon SEB stimulation. Therefore, we cannot exclude the possibility that lymphocytes become more susceptible to apoptosis upon stimulation in response to the HIIT session. However, it would be expected that an increased susceptibility to apoptosis upon stimulation affects both SEB and PHA proliferative responses, but a decrease in cell proliferation related to the HIIT session was observed only in response to SEB stimulation, thus suggesting that mechanisms other than reduced cell viability would explain our findings.

Considering that early lymphocyte activation by SEB might be altered in response to the HIIT session, leading to reduction in proliferation, we evaluated the effect of exercise on the frequency of CD25^+^ and CD69^+^ cells upon SEB stimulation. Our analysis initially indicated that SEB-activation of CD4^+^ and CD19^+^, but not CD8^high^ lymphocytes, was reduced after HIIT. However, the HIIT session also promoted a shift in the lymphocyte balance, with a 40–50% reduction in the frequency of CD4^+^ and CD19^+^ cells. When corrected for the percentage of CD4 and CD19, no effect of HIIT was observed on the percentage of CD25^+^ and CD69^+^ cells, suggesting that early lymphocyte responsiveness to superantigenic stimulation was not altered by exercise, despite the reduced proliferative response.

The reduced proliferative response to SEB stimulation, despite the absence of an effect of the HIIT session on early activation marker expression, could be explained by the proliferation assay employed. When a constant number of PBMC is used to evaluate lymphocyte proliferation, alterations in the proportion of responding and non-responding cells in the culture can affect the results. Acute exercise causes a redistribution of lymphocyte subsets in the circulation such that relative percentages of different subsets are altered, in particular, the proportion of natural killer (NK) cells to T lymphocytes [48]. NK cells do not proliferate in response to superantigenic/mitogenic stimulation in culture [49]. Thus, changes in relative composition of lymphocytes are suggested to be the most probable contributing factor to the reduction in mitogen response after exercise [9]. The HIIT promoted a redistribution of lymphocytes, altering the relative percentages of lymphocyte subsets. Although we did not use classical markers for quantification of NK cells (CD56 and CD16), our data suggest that the reduction in the frequency of CD4^+^ and CD19^+^ lymphocytes has happened along with an increased proportion of NK cells after HIIT, considering the frequency of CD8^low^ cells (data not shown). Considering that NK cells do not proliferate in response to superantigen stimulation and that there were proportionally fewer T cells capable of responding to the superantigenic stimulation in our assay, a lower proliferation response was found. Supporting this hypothesis, we observed a higher percentage of cells in the M1 region of CFSE histograms after the HIIT session, indicating that a higher proportion of cells did not proliferate in response to superantigenic stimulation. We also observed an higher concentration of IL-2 in culture supernatants of SEB-stimulated cells. This observation may reflect a reduced IL-2 consumption by cells, which is in accordance with the lower proliferative response. However, because no effect of the HIIT was observed on the proliferation and IL-2 supernatant concentration in response to PHA stimulation (PHA also does not induce NK proliferation), modification of the proportion of antigenic/mitogenic responsive cells does not completely explain our findings. PHA- and SEB-induced T cell activation are mechanistically different, and sustained lymphocyte proliferation in response to SEB is dependent on later T cell activation (HLA-DR expression) and cell-cell contact mediated by LFA-1 [50]. Therefore, it is possible that HIIT effects on SEB-induced proliferative response may involve modulation of later T cell activation mechanisms not investigated in our study. Also, different from SEB, induction of lymphocyte proliferation by PHA is only moderately dependent on the function of accessory cells [51]. Therefore, HIIT-induced alterations in monocyte and NK cell function, two important antigen-presenting cells involved in lymphocyte activation by SEB [52], could influence the magnitude of any subsequent proliferative response.

Our findings reported here has brought to light some unanswered issues that deserve further investigation. It will be of great interest to evaluate, for example, whether restoring the reducing capacity of lymphocytes after HIIT, *in vitro* or *in vivo*, by antioxidant supplementation, also restores the proliferative response. It will be equally relevant to determine the duration of the effect of HIIT in lymphocytes. Because of sample limitation we were not able to evaluate HIIT effect on lymphocyte proliferation and redox imbalance at later time points. And because T cell function is central to cellular immunity, and one consequence of inefficient T cell activation is increased susceptibility to infection [53], it will be important to access how lymphocyte function modulation by an acute HIIT session can affect individual immunity and susceptibility to infection. On the other hand one can speculate that HIIT effect on lymphocyte proliferative response can be beneficial in the context of chronic inflammatory diseases. As recently demonstrated, a HIIT training program resulted in improvement of inflammatory markers and cardiac risk factors in individuals with rheumatoid arthritis and juvenile idiopathic arthritis [54]. Reduced lymphocyte activity in response to HIIT may represent an additional benefit of this training modality to rheumatoid arthritis patients.

In summary, our findings show that a single bout of HIIT promotes lymphocyte oxidative stress and reduces superantigen-induced proliferation, but not mitogen-induced proliferation, what cannot be explained by alteration of the early lymphocyte activation response to superantigen.

## Supporting Information

S1 Dataset Raw data of the study. (XLSX)
